# Long-Term Suppression of Circulating Proinflammatory Cytokines in Multiple Sclerosis Patients Following Autologous Haematopoietic Stem Cell Transplantation

**DOI:** 10.3389/fimmu.2021.782935

**Published:** 2022-01-19

**Authors:** Kevin Hendrawan, Melissa L. M. Khoo, Malini Visweswaran, Jennifer C. Massey, Barbara Withers, Ian Sutton, David D. F. Ma, John J. Moore

**Affiliations:** ^1^ Blood, Stem Cells and Cancer Research Programme, St Vincent’s Centre for Applied Medical Research, Darlinghurst, NSW, Australia; ^2^ St Vincent’s Clinical School, Faculty of Medicine, University of New South Wales, Sydney, NSW, Australia; ^3^ Department of Neurology, St Vincent’s Hospital, Darlinghurst, NSW, Australia; ^4^ Department of Haematology, St Vincent’s Hospital, Darlinghurst, NSW, Australia

**Keywords:** cytokines, AHSCT, multiple sclerosis, T helper 17, long-term suppression

## Abstract

Autologous haematopoietic stem cell transplantation (AHSCT) is a therapeutic option for haematological malignancies, such as non-Hodgkin’s lymphoma (NHL), and more recently, for autoimmune diseases, such as treatment-refractory multiple sclerosis (MS). The immunological mechanisms underlying remission in MS patients following AHSCT likely involve an anti-inflammatory shift in the milieu of circulating cytokines. We hypothesised that immunological tolerance in MS patients post-AHSCT is reflected by an increase in anti-inflammatory cytokines and a suppression of proinflammatory cytokines in the patient blood. We investigated this hypothesis using a multiplex-ELISA assay to compare the concentrations of secreted cytokine in the peripheral blood of MS patients and NHL patients undergoing AHSCT. In MS patients, we detected significant reductions in proinflammatory T helper (Th)17 cytokines interleukin (IL)-17, IL-23, IL-1β, and IL-21, and Th1 cytokines interferon (IFN)γ and IL-12p70 in MS patients from day 8 to 24 months post-AHSCT. These changes were not observed in the NHL patients despite similar pre-conditioning treatment for AHSCT. Some proinflammatory cytokines show similar trends in both cohorts, such as IL-8 and tumour necrosis factor (TNF)-α, indicating a probable treatment-related AHSCT response. Anti-inflammatory cytokines (IL-10, IL-4, and IL-2) were only transiently reduced post-AHSCT, with only IL-10 exhibiting a significant surge at day 14 post-AHSCT. MS patients that relapsed post-AHSCT exhibited significantly elevated levels of IL-17 at 12 months post-AHSCT, unlike non-relapse patients which displayed sustained suppression of Th17 cytokines at all post-AHSCT timepoints up to 24 months. These findings suggest that suppression of Th17 cytokines is essential for the induction of long-term remission in MS patients following AHSCT.

## Highlights:

1. We report significant changes in some of the blood cytokines in MS compared to NHL patients following AHSCT.
2. Suppression of Th17 proinflammatory cytokines is crucial for long term disease remission in MS patients following AHSCT.

## Introduction

While autologous haematopoietic stem cell transplantation (AHSCT) is a successful treatment option for certain haematological malignancies (1–3), including non-Hodgkin’s lymphoma (NHL) (4, 5), it has also been demonstrated to be an effective treatment for autoimmune diseases, such as multiple sclerosis (MS) when compared to other disease modifying therapies (6). A central hypothesis for the observed disease remission is that AHSCT ‘resets’ the patient’s presumably autoreactive immune system to a more tolerogenic state through conditioning chemotherapy and infusion of ‘rescue’ autologous haematopoietic stem cells (7–13). It has been proposed that resetting of the immune balance and remission from CNS inflammation occurs through the removal of proinflammatory cells and the regeneration of immunoregulatory cells that are important in maintaining peripheral tolerance (9). These cells are known to exert a myriad of immunoregulatory mechanisms, including the suppression of proinflammatory cytokine secretion (14–16).

Cytokines are pleiotropic extracellular proteins secreted by immune, endothelial, and stromal cells which predominantly function to coordinate immune responses. Cytokines can be categorised into several classes depending on their functions. For instance, proinflammatory cytokines are those that promote immune responses, such as those involved in T helper (Th) 17-related immune responses, including interleukin (IL)-17 (17), IL-23, IL-21, IL-6, IL-8 (18) and IL-1β (19), and those involved in Th1-related immunity, such as TNFα (20), IFNγ (21) and IL-12p70 (22). In contrast, certain cytokines suppress immune responses, such as IL-10 (23). In the Th1/Th2 differentiation paradigm, IL-4 is known to induce Th2 differentiation, promoting humoral immune responses (24) and suppresses Th1-related responses (25). Because Th1 responses are strongly correlated to MS pathogenesis (26, 27) and Th2 responses are associated with the dampening of MS activity (28), IL-4 is considered an anti-inflammatory cytokine in MS. Additionally, IL-2 is involved in the proliferation and homeostasis of T cells (29) and can also be considered anti-inflammatory due to its important role in the differentiation of immunoregulatory T regulatory cells (Tregs) (30–32).

In MS pathogenesis, the dysregulation of cytokines has been reported to correlate with disease activity (26, 33, 34). A meta-analysis evaluating Th17 cell status and Th17 related cytokines in the peripheral blood of MS patients revealed that the proportion of Th17 cells, and the levels of IL-17 and IL-23 were increased compared to control subjects (34), strongly suggesting that Th17-related responses contributes to MS pathogenesis. Indeed, IL-17-producing mucosal associated invariant T (MAIT) cells were detected around actively demyelinating lesions in post-mortem brain sections of MS patients (35), supporting this hypothesis. Additionally, Th1-related cytokines are also known to contribute to MS pathogenesis (36). In regards to anti-inflammatory cytokine profiles, the majority of studies report significantly lower levels of IL-10-producing regulatory cells in the circulation of relapsing-remitting MS patients (37, 38), whilst one study analysing progressive MS patients reported elevated IL-10 serum levels, along with higher levels of TNFα and IFNγ (39). Altogether these studies denote a dysregulated coordination of the immune system in MS patients.

Thus far, only two studies have investigated the evolution of the cytokine milieu in the blood of MS patients following AHSCT (40, 41), with both employing a lymphoablative cyclophosphamide chemotherapy regimen. Wiberg and colleagues (40) demonstrated significant but transient changes to cytokines predominantly associated with innate immunity following AHSCT that returned to baseline levels by the 3 month timepoint. Jaime-Perez and colleagues (41) reported significant reductions in proinflammatory cytokines IL-21 and IL-22, and increased levels of anti-inflammatory cytokines CCL2 and CCL4 at 14 days following transplantation without longer term follow-up. Overall, these studies give little indication of the influence of AHSCT on adaptive immunity. Furthermore, these studies failed to address the degree to which the cytokine shifts relate to early chemotherapy-induced lymphopenia as opposed to a disease specific consequence of immune reconstitution, which likely occur at later timepoints post-transplantation.

The BEAM conditioning regimen used in AHSCT for MS patients is also widely used in AHSCT for NHL, thus making NHL patients an ideal comparator group to investigate cytokine changes post-AHSCT. As NHL is characterised by a defect in the early development of lymphocytes (42), aberrant cytokine expression is also evident in this disease. Abnormal levels of several cytokines in the patient blood have been associated with NHL pathology, including IL-2, IL-8, IL-10, IFNγ and TNF (43, 44). However, the most consistent finding is significantly elevated IL-6 in NHL patients compared to healthy controls (44–46). In one study, gene expression of IL-10, TNFα, and IL-8 was significantly increased in the blood of NHL patients at day 15-30 post-AHSCT (47), supporting the hypothesis that AHSCT induces cytokine changes that may alter the immune system. Other studies in the allogeneic setting have also reported changes to the cytokine milieu of NHL patients following stem cell transplantation (48–52).

Here, we sought to evaluate durable changes in the peripheral blood cytokine profile of MS patients following AHSCT with a myeloablative conditioning chemotherapy, BEAM + Horse ATG (20mg/kg/day). Globally, this is the second-most used conditioning regimen in AHSCT for MS. Given MS pathogenesis associates with an adaptive immune response, we focus specifically on pathogenic adaptive immune-related inflammatory cytokines. We hypothesise that proinflammatory cytokines will be suppressed and anti-inflammatory cytokines will be increased post-AHSCT, reflecting the tolerogenic shift as indicated by immune reconstitution data (8, 35, 53). To further determine the degree to which AHSCT re-establishes immune tolerance, a comparator arm of NHL patients has been recruited. This cohort exhibit different immunological abnormalities to MS and received AHSCT as treatment, albeit with a slightly different chemotherapy regimen (BEAM only) (53). Inclusion of the NHL comparator arm enables differentiation between changes induced by chemotherapy versus immune reconstitution in the autoimmune setting, which has not been previously reported but is critical for understanding the mechanisms underlying disease remission post-AHSCT.

## Methods

### Study Cohorts and Sample Collection

Study cohorts consisted of MS and NHL patients undergoing AHSCT [as described previously (53)], as well as healthy controls age and sex-matched to the MS cohort after informed written consent and institutional ethics committee approval according to the Declaration of Helsinki (Ethics ID: HREC SVH File No. 10/135) (**Table 1**). NHL patients were recruited from the same transplant unit during the same period as the MS patients and similarly received BEAM conditioning but did not receive ATG. MS patients in this study were enrolled as part of a Phase II clinical trial of AHSCT for MS, (which is registered with the Australian New Zealand Clinical Trials Registry, ACTRN12613000339752), as described previously (53). Patients were referred by their primary neurologist. Eligible patients were aged 18-60 years, with a diagnosis of MS according to the McDonald criteria (54) and Kurtze Expanded Disability Status Scores (55) (EDSS) 2-7. The transplantation regimen has been described previously (53). Prior to AHSCT, MS patients have discontinued disease modifying therapies (DMTs) for at least 4 weeks prior to specimen collection. The time points for peripheral blood specimen collection were pre-AHSCT, day 8 and day 14, and 3 months, 6 months, 1 year and 2 years post-AHSCT. Patients were also monitored for any clinical developments following AHSCT, such as serum sickness at the early timepoints, secondary autoimmunity, and relapses post-AHSCT. Relapse was defined as the detection of new magnetic resonance imaging (MRI) lesions (56) and/or occurrence of clinical relapse (54) post-AHSCT. EDSS progression was not accounted for in determining treatment response due to the differing underlying pathogenesis. Unblinding of patient response data towards AHSCT occurred after the completion of experiments. Due to logistical challenges associated with interstate patient relocations, the NHL cohort was only followed up until 1-year post-AHSCT.

**Table 1:** Study cohort characteristics.

Characteristics	Tx cohorts		Non-Tx cohorts
	MS (n = 22)	NHL (n = 9)	HC (n = 9)
*Age/age at transplantation*	36.5 (22-55)	60 (29-68)	36 (25-52)
*Female*	13 (59%)	5 (56%)	5 (56%)
*Previous DMT:*			
2 to 3	14 (63%)		
4 to 5	8 (37%)		
*Previous exposure to natalizumab*	15 (68%)		
*EDSS:*			
<4	8 (36%)		
4 to 6	8 (36%)		
<6	6 (27%)		

Data are median (range) or n(%) unless stated otherwise.

DMT, disease modifying therapies; EDSS, Expanded Disability Status Scale; Tx, Transplantation.

MS, Multiple Sclerosis; NHL, non-Hodgkin’s lymphoma; HC, Healthy Controls

Study cohort characteristics.

### Peripheral Blood Cytokine Detection

Platelet-poor plasma (PPP) was isolated and preserved from 20mL peripheral blood specimens collected in EDTA tubes from study participants. Specimens were processed within 12-24 hours of collection by centrifugation (400g for 10 minutes), removal of platelet-rich plasma, and further centrifugation (1500g for 20 minutes). The resulting PPP supernatant was aspirated and aliquoted into tubes for storage at -80°C until use. Vials of PPP were freshly thawed on the day of the cytokine detection assay. The cytokines IL-17, IFNγ, TNFα, IL-12p70, IL-1β, IL-10, IL-2, IL-6, IL-8, IL-4, IL-23, and IL-21 were quantified in patient and healthy control PPP using a Human High Sensitivity T cell Magpix kit (Millipore, cat # MPHSTCMAG28SK13) according to manufacturer’s protocols.

PPP samples from patients and healthy controls were thawed, mixed well by vortex, and centrifuged for 5 minutes at 10,000 rpm to remove plasma particulates that can affect detection. Samples undergo a maximum of 2x freeze/thaw cycles. Cytokine detection antibody beads, background serum matrix, standards, quality controls, and a 96-well detection plate were provided by the kit and prepared prior to the experiment. 25μL of each standard and quality controls were added into designated wells in the plate. The background well was considered a blank well in which nothing was added. For the analysis of plasma samples, serum matrix was added into the background, standard and quality control wells. 25μL of each PPP sample was then added into designated sample wells. 25μL of the detection bead mixture was then added into the background, standard, control, and sample wells. The plate was then sealed with a plate sealer, wrapped in foil to protect from light and incubated with agitation on a plate shaker at 500 rpm for 16-18 hours. Cytokine concentrations were then quantified by detecting plate luminescence using a Luminex MAGPIX detection system (Merck Millipore).

In the first assay run, samples, standards, and quality controls were run in duplicate, as per manufacturer’s instructions, to assess the reproducibility of detection within each well and account for intra-assay variation. However, running duplicate wells limit the number of samples quantified per plate. This may lead to longitudinal timepoint samples from a single patient to be quantified between two or more experimental plates. Such analysis of longitudinal samples introduces inter-assay variation that could affect biological measurements across treatment timepoints from a single patient. Therefore, subsequent assays were run in single wells, as others have done (57, 58), because the coefficient of variation between duplicate wells from the first experiment were between 1-5%. This allowed more space to run all longitudinal timepoints from a single patient within the same plate, minimising inter-assay variation.

### Statistical Analysis

The statistical difference between data collected from healthy controls and both disease cohorts at pre-AHSCT was analysed using a Mann-Whitney U test and the Bonferroni *post-hoc*. For the analysis of data from transplantation studies, the statistical difference between individual post-AHSCT timepoints in MS or NHL patients was compared to pre-AHSCT timepoint using the following methods.

Firstly, the data was analysed using a one-way repeated measure ANOVA in Graphpad Prism V8 (equivalent of a linear mixed model analysis). This analysis was performed over the non-parametric Wilcoxon’s test or Friedman’s test as it is a robust method that allows inclusion of patients with missing timepoint data. The repeated measures ANOVA was performed in conjunction with Holm-Sidak *post-hoc* analysis and assessment of data residuals. If the data residuals did not conform to the linear model, the data was transformed using natural log method and re-analysed using the repeated measures ANOVA. If the data residuals still did not conform to the linear model, then the timepoint data were analysed using Wilcoxon’s test with Bonferroni *post-hoc* tests. During the analysis of relapsed MS patients, the day 8 (n=1) and day 14 (n=2) post-AHSCT timepoints were excluded due to low sample sizes.

Blood cytokine concentrations between relapsed and non-relapsed MS patients were compared at each timepoint (pre-AHSCT, and at day 8, day 14, 3 months, 6 months, 12 months, and 24 months post-AHSCT) using a two-way repeated measures ANOVA in conjunction with Sidak *post-hoc* analysis and assessment of data residuals. Similarly, if the data residuals did not conform to the linear model, the data was transformed using the natural log method and re-analysed. These analyses are consistent with previous methods (35, 59).

## Results

### T Cell-Associated Proinflammatory Cytokines Were Suppressed Within First 3 Months Post-AHSCT in MS Patients

Firstly, the cytokine profile of MS patients prior to AHSCT was assessed (**Table 2**). When compared to plasma cytokine concentrations within healthy controls, only the proinflammatory cytokines IL-17 and IFNγ were significantly increased in the MS cohort pre-AHSCT. In contrast, except for IL-4, IL-1β, IL-6, IL-8 and TNFα, pre-AHSCT NHL patients exhibited significantly lower levels of all other cytokines than healthy controls and pre-AHSCT MS patients. Additionally, levels of IL-4 and IL-1β were only significantly reduced in NHL patients when compared to MS patients at pre-AHSCT.

**Table 2:** Comparison of plasma cytokine levels between MS and NHL patients before transplantation and healthy controls.

Cytokines	MS patients (n = 22)	NHL patients (n = 9)	Healthy controls (n = 9)	Statistical analysis (Man-Whitney U test)		
				MS vs HC	MS vs NHL	NHL vs HC
IL-17 (pg/mL)	18.6 ± 2	6.6 ± 1.1	10.72 ± 1.1	*p*=0.006	*p*<0.0001	*p*<0.006
IFNγ (pg/mL)	18.03 ± 2.3	5.7 ± 1.3	11.39 ± 1.4	*p*=0.014	*p*<0.0001	*p*<0.004
IL-10 (pg/mL)	56.57 ± 9.2	21.99 ± 5.1	47.37 ± 5.7	NS	*p*=0.0025	*p*=0.01
IL-12p70 (pg/mL)	5.55 ± 0.93	2.3 ± 0.4	4.4 ± 0.6	NS	*p*=0.002	*p*=0.004
IL-2 (pg/mL)	4.5 ± 0.8	1.44 ± 0.3	4 ± 0.45	NS	*p*=0.0007	*p*=0.0005
IL-21 (pg/mL)	8.6 ± 1.7	2.6 ± 0.6	6.8 ± 1.1	NS	*p*=0.0006	*p*=0.003
IL-4 (pg/mL)	86.79 ± 18.19	24.7 ± 8.6	71.32 ± 21.46	NS	*p*=0.002	NS
IL-23 (pg/mL)	653.3 ± 104.9	193.5 ± 45.7	642.2 ± 114.2	NS	*p*=0.0004	*p*=0.0008
IL-1β (pg/mL)	3 ± 0.48	0.97 ± 0.2	1.81 ± 0.34	NS	*p*=0.0004	NS
IL-6 (pg/mL)	4.3 ± 1.7	2 ± 0.6	8.2 ± 3.73	NS	NS	NS
IL-8 (pg/mL)	6.2 ± 1.35	3.5 ± 0.4	7.83 ± 2.2	NS	NS	NS
TNFα (pg/mL)	6 ± 0.5	8.6 ± 1.2	5.5 ± 0.5	NS	NS	NS

Data are presented as means ± standard error.

IFN, interferon; IL, interleukin; TNF, Tumor necrosis factor; MS, Multiple sclerosis.

A P-value lower than 0.017 is considered significant after corrected for multiple comparisons using the Bonferoni post-hoc.

Next, we investigated early cytokine changes in our MS cohort up to 3 months post-AHSCT, where substantial cytokine shifts have previously been reported (40). Additionally, because AHSCT is known to alter the cytokine profile of NHL patients (47), we also investigated cytokine changes in NHL patients undergoing AHSCT to compare the effect of our transplant regimen to previously reported regimens and determine any possible diseases specific cytokine changes following AHSCT.

**Figure 1:**
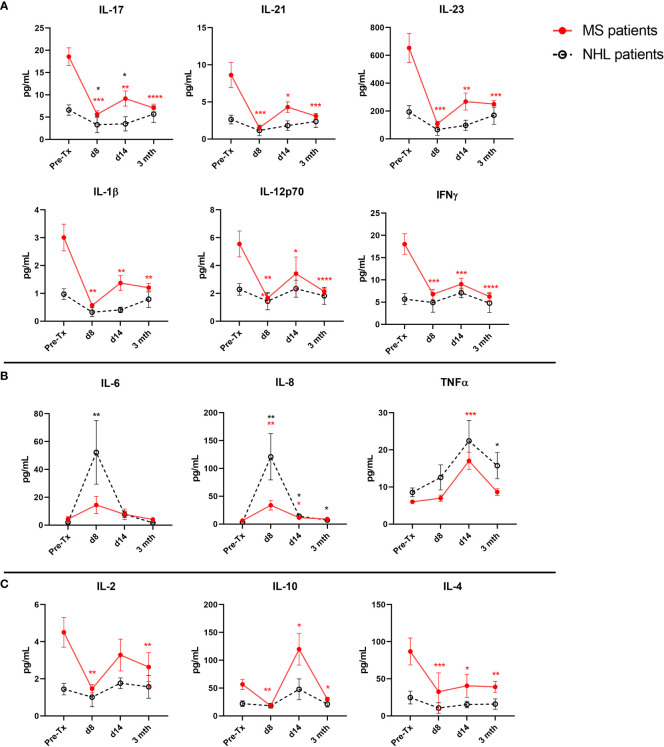
Early post-AHSCT changes to plasma cytokine levels in MS and NHL patients. Cytokine concentrations were quantified at pre-AHSCT (pre-Tx), day 8 (d8), day 14 (d14), and 3 months (mth) post-AHSCT. **(A)** Immediate suppression of several T cell-associated proinflammatory cytokines were observed early after transplantation (Th17: IL-17, IL-21, IL-23, and IL-1β; Th1 IL-12p70 and IFNγ) in MS patients. **(B)** Some proinflammatory cytokines exhibit early surges post-transplantation (IL-8, IL-6 and TNFα) in both MS and NHL patients. **(C)** Plasma concentration of anti-inflammatory cytokines (IL-10, IL-4, and IL-2) at specified timepoints in patients undergoing AHSCT. MS cohort sample size is n=22, and NHL cohort sample size is n=9. Sample sizes per timepoint are as follows: 1) Pre-Tx: MS n=22; NHL n=9 2) d8: MS n=9; NHL n=5, 3) d14: MS n=10; NHL n=5, and 4) 3 months: MS n=21; NHL n=9. Analysis was performed using a one-way repeated measures ANOVA with Holm-Sidak *post-hoc*. *p < 0.05; **p < 0.01; ***p < 0.001; ****p < 0.0001.

At baseline MS patients have elevated levels of all pro-inflammatory cytokines when compared with NHL patients undergoing AHSCT (**Figure 1A**). We observe significant reductions in two Th1 (IFNγ and IL-12p70) and four Th17 (IL-17, IL-21, IL-23, and IL-1β) proinflammatory cytokines as early as day 8 post-AHSCT in the MS cohort, with persistent reduction in these cytokines out to 3 months. Generally, pro-inflammatory cytokine levels were stable across the NHL patients in the early post-transplant period, though a significant reduction in IL-17 at day 8 and day 14 post-AHSCT was noted when compared to pre-AHSCT samples.

Some proinflammatory cytokines exhibited transient spikes during the early immune reconstitution period (**Figure 1B**). IL-8 and IL-6 levels rose sharply in NHL patients, and to a lesser degree, MS patients (IL-8), trending back to baseline by 3 months. TNFα reached its peak 14 days post-AHSCT, returning to pre-AHSCT levels in MS patients by 3 months.

Dynamic changes were also observed in plasma anti-inflammatory cytokine levels in MS patients following AHSCT (**Figure 1C**). IL-10 dramatically increased from baseline at day 14 post-AHSCT in MS patients. By 3 months post-AHSCT, IL-10 decreased to levels that were significantly lower than baseline in MS patients. IL-2 and IL-4 were significantly reduced at day 8 and 3 months post-AHSCT compared to baseline in MS patients. No significant changes to anti-inflammatory cytokines were observed in NHL patients post-AHSCT.

### T Cell-Associated Proinflammatory Cytokines Remain Suppressed up to 24 Months post-AHSCT in MS Patients

**Figure 2:**
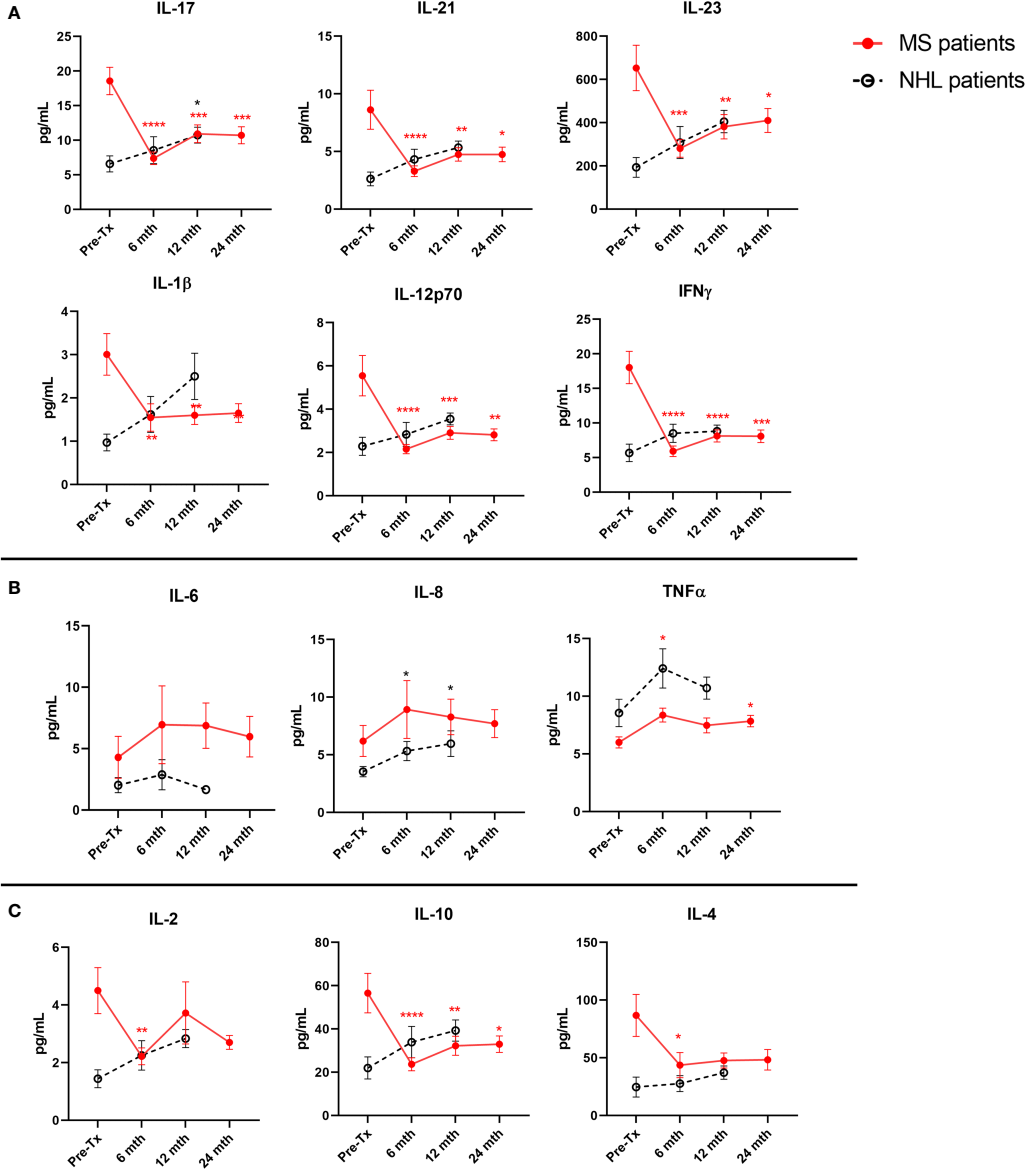
Long-term post-AHSCT changes to plasma cytokine levels in MS and NHL patients. Cytokine concentrations were quantified at pre-AHSCT (pre-Tx), 6 months (mth), 12 months, and 24 months post-AHSCT. **(A)** Suppression of T cell associated proinflammatory cytokines (Th17: IL-17, IL-21, IL-23, and IL-1β; Th1 IL-12p70 and IFNγ) are sustained up to 24 months post-AHSCT in MS patients only. **(B)** In contrast, the initial increase in other proinflammatory cytokines (IL-8, IL-6 and TNFα) were not maintained at later timepoints. **(C)** Plasma concentration of anti-inflammatory cytokines (IL-10 and IL-2) at specified timepoints in patients undergoing AHSCT. MS cohort sample size is n=22, and NHL cohort sample size is n=9. Sample sizes per timepoint are as follows: 1) 6 months: MS n=21; NHL n=9, 2) 12 months: MS n=21; NHL n=9, and 3) 24 months: MS n=21. Analysis was performed using a one-way repeated measures ANOVA with Holm-Sidak *post-hoc*. *p < 0.05; **p < 0.01; ***p < 0.001; ****p <0.0001.

Both Th1 (IFNγ and IL-12p70) and Th17 (IL-17, IL-21, IL-23, and IL-1β) proinflammatory cytokines were significantly suppressed at all long-term timepoints (6, 12, and 24 months) examined in MS patients when compared to baseline (**Figure 2A**). The only change detected in T cell-associated cytokines in NHL patients was a significant increase in IL-17 at 12 months post-AHSCT compared to baseline. In contrast, both the proinflammatory cytokines IL-8 and IL-6 were not significantly different to pre-AHSCT levels at 6 months, 12 months, and 24 months post-AHSCT in MS patients (**Figure 2B**). However, in NHL patients IL-8 was found to be significantly higher than baseline levels at 6 months and at 12 months post-AHSCT, while no changes were detected for IL-6. TNFα remained stable at 6 months and at 12 months post-AHSCT compared to baseline in NHL patients, whereas in MS patients TNFα was significantly increased at 6 months and 24 months post-AHSCT. Interestingly, the anti-inflammatory cytokine IL-10 was significantly reduced at 6 months, 12 months, and 24 months post-AHSCT compared to baseline levels in MS patients (**Figure 2C**), whereas both IL-4 and IL-2 were only significantly reduced at 6 months post-AHSCT. No significant changes were observed in the blood concentrations of IL-10, IL-4, and IL-2 in NHL patients post-AHSCT.

### Elevated Th17 Proinflammatory Cytokines Were Associated With Relapse in MS Patients Post-AHSCT

In general, most MS patients responded well to AHSCT, with only 4 out of 22 patients experiencing disease relapse between 11 months to 23 months post-AHSCT (**Supplementary Table 1**). Only one patient within the non-relapsed cohort developed secondary autoimmunity (microscopic colitis) at 2 months post-AHSCT and this was reflected in a brief elevation in the concentration of cytokines at 3 months, such as IFNγ and IL-10. This patient was treated with oral budesonide and recovered.

**Figure 3:**
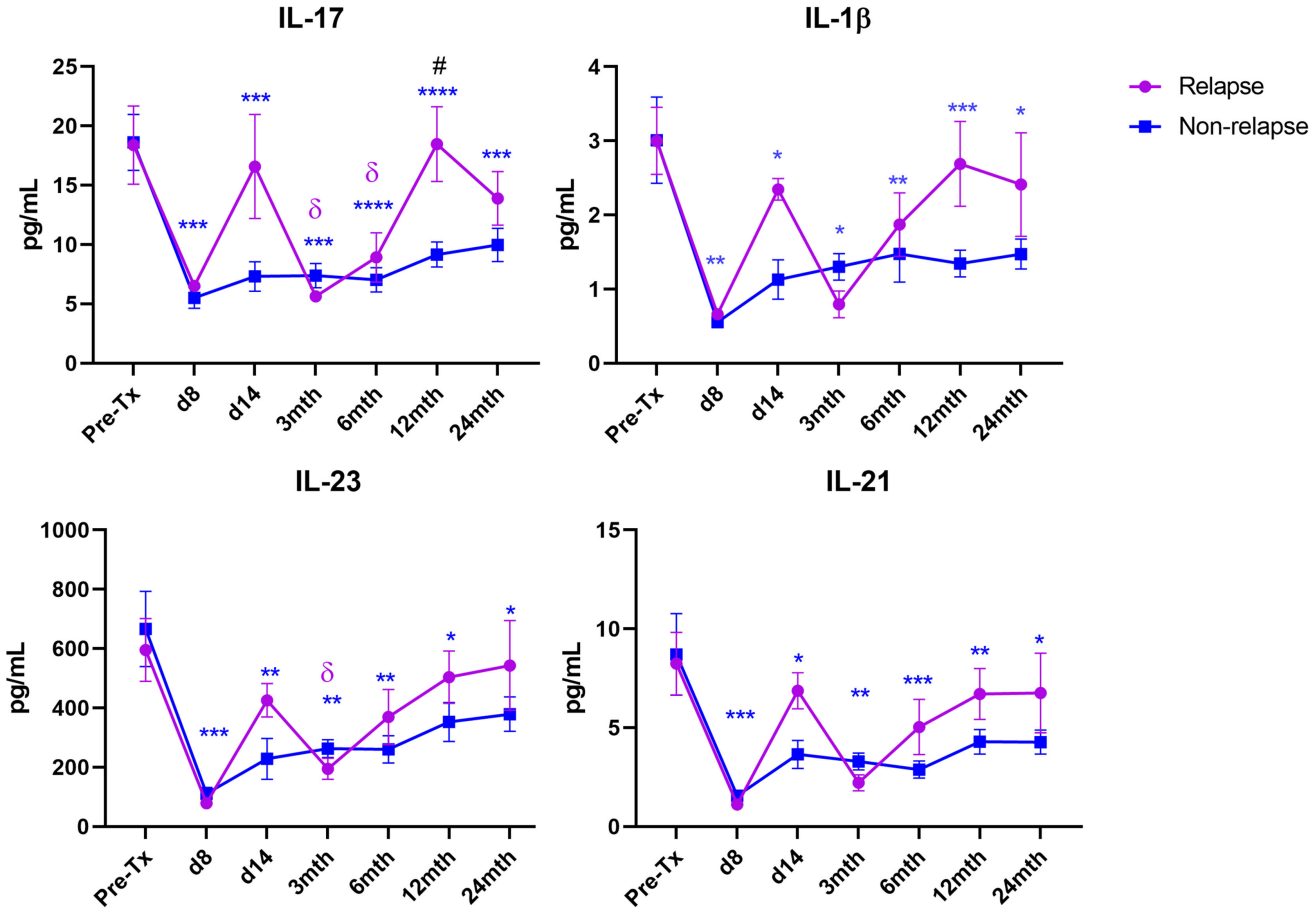
Th17 cytokine levels in relapse and non-relapse MS patients following AHSCT. Cytokine concentrations were quantified at pre-AHSCT (pre-Tx), day 8 and day 14, and at 3 months (mth), 6 months, 12 months, and 24 months post-AHSCT in relapsed (n=4) and non-relapsed patient cohorts (n=18). Sample sizes per timepoint within each cohort are as follows: 1) Pre-Tx: Relapsed n=4; Non-Relapsed n=18, 2) Day 8: Relapsed n=1; Non-Relapsed n=8, 3) Day 14: Relapsed n=2; Non-Relapsed n=8, 4) 3 months: Relapsed n=4; Non-Relapsed n=17, 5) 6 months: Relapsed n=4; Non-Relapsed n=17, 6) 12 months: Relapsed n=4; Non-Relapsed n=17, 7) 24 months: Relapsed n=4; Non-Relapsed n=17. Comparison of mean cytokine concentrations between relapsed and non-relapse cohorts at each timepoint was performed using a two-way repeated measures ANOVA with Sidak *post-hoc*. Comparison of each post-AHSCT timepoint to Pre-Tx was performed using a one-way repeated measures ANOVA with Holm-Sidak *post-hoc*. Symbols represent significant difference between pre-AHSCT and a post-AHSCT timepoint in each cohort. Non-Relapsed cohort: *p < 0.05; **p < 0.01; ***p < 0.001; ****p < 0.0001. Relapsed cohort: ^δ^ *p* < 0.05. ^#^(*p* < 0.05) represents a significant difference between the relapse and the non-relapse cohort at each timepoint.

MS patients who experienced disease relapses post-AHSCT exhibited distinct post-AHSCT blood Th17 cytokine profiles compared to patients who remained free of relapse (**Figure 3**). Non-relapse patients exhibited sustained suppression of the Th17 cytokines IL-17, IL-23, IL-21 and IL-1β at all post-AHSCT timepoints up to 24 months. In contrast, these cytokines generally show no significant changes post-AHSCT in relapsed patients, except for IL-17, which was significantly decreased at 3 months and 6 months, and IL-23, which was significantly decreased at 3 months. Of note, IL-17 was significantly increased in relapsed patients compared to the non-relapsed cohort at 12 months post-AHSCT.

Elevated levels of circulating proinflammatory cytokines were previously reported to be a biomarker of serum sickness as a result of the transplantation regimen (60). Therefore, we investigated the possibility that patient response post-AHSCT is determined by serum sickness. Importantly, the differences observed between MS patients with different responses to AHSCT in this study was not significantly correlated to serum sickness resulting from treatment (data not shown).

## Discussion

This is the first study to report sustained and profound changes to T cell-related proinflammatory cytokines in MS patients up to 24 months post-AHSCT. It is also the first to report these cytokine changes in comparison to a non-autoimmune disease cohort undergoing similar therapy. Few studies have examined the evolution of the cytokine milieu in patients with autoimmune diseases post-AHSCT (40, 41, 61, 62). These studies described significant reductions in the cytokine levels in the blood of both systemic sclerosis (62) and MS (40, 41) patients early post-AHSCT. However, these changes were transient and the overall cytokine concentrations were reported to return to baseline levels by 3-12 months post-AHSCT. Here, we have shown that i) proinflammatory Th17 and Th1 cytokines are elevated in MS patients pre-AHSCT compared to healthy controls; ii) significant and sustained reductions in proinflammatory Th17 and Th1 cytokines up to 24 months post-AHSCT that are specific to MS patients and not NHL patients; and iii) IL-17 was significantly elevated at 12 months post-AHSCT in the relapsed patients compared to non-relapse patients for MS.

Consistent with previous findings (33, 63, 64), the proinflammatory cytokines IL-17 and IFNγ were significantly elevated in the blood of MS patients pre-AHSCT when compared to healthy controls. Other cytokines investigated here, including the anti-inflammatory cytokines, were not significantly different between pre-AHSCT MS patients and healthy controls. A significant proportion of these cytokines were significantly reduced in NHL patients at pre-AHSCT compared to healthy controls and MS patients. These reductions are likely attributed to rituximab pre-treatment within the NHL cohort, which has previously been shown to significantly reduce cytokine levels in patients (65), and was not used in NHL patients. Indeed, only one MS patient in our cohort was pre-treated with rituximab (**Supplementary Table 1**), and their cytokine concentrations were comparable to the NHL cohort (data not shown), which supports this hypothesis.

Following AHSCT, both Th17-related cytokines (IL-17, IL-1β, IL-21 and IL-23) and Th1-related cytokines (IFNγ and IL-12p70) were significantly reduced in MS patients, and this change was sustained up to 24 months post-AHSCT. In contrast, these proinflammatory cytokines show no-significant changes in NHL patients post-AHSCT. Considering the low starting levels of these cytokines in NHL patients at pre-AHSCT, the post-transplant evolution of these cytokines was generally similar across both disease cohorts. These findings highlight the immunosuppressive action of the conditioning regimen in AHSCT, which was proposed as an important aspect in inducing disease remission for both lymphomas and autoimmune diseases (7). Hence, the suppression of proinflammatory cytokines may simply reflect the ablation of immune cell populations following the conditioning regimen. However, in our MS cohort, total lymphocyte recovery was reached by 12 months post-AHSCT (53), whilst proinflammatory cytokines remain low for a further 12 months after this. This finding supports the hypothesis that AHSCT “recalibrates” the dysregulated immune system in MS patients beyond simple induction of lymphopenia. Long-term suppression of proinflammatory cytokines post-AHSCT may not be observed in previous reports of AHSCT in MS because the patients in these studies received non-myeloablative conditioning (40, 41), unlike this study, which used a higher intensity myeloablative BEAM conditioning.

One point of difference between the MS and NHL group was that ATG was used in the conditioning regimen of the MS group but not in the NHL group (53). ATG is a potent T cell suppressive agent that has been demonstrated to significantly reduce the gene expression of several cytokines (66). This may explain the increase in IL-6 observed at day 8 post-AHSCT in NHL patients that was not observed in MS patients. It is also noteworthy that the NHL patients were older than both the MS cohort and the healthy controls as previously highlighted (53) and this may therefore impact post-AHSCT cytokine evolution.

A few proinflammatory cytokines (IL-8, IL-6 and TNFα) were comparable between healthy controls and both disease cohorts prior to AHSCT. Although the long-term profiles of these cytokines were essentially unchanged or minimally changed from baseline, they appear to play a more important role in the early reconstitution process. All three cytokines increased in the early post-transplant period in NHL treated patients, and IL-8 and TNFα, which similarly increased early post-AHSCT in both NHL and MS patients. IL-8 is a proinflammatory cytokine associated with acute phase immune responses that is produced by innate immune cells when stimulated by TNFα. TNFα is a macrophage derived cytokine that is well established to play an important role in the control of viral infection (67), which are common post-transplantation (68, 69). Hence, the significant surges in IL-8 and TNFα a few days post-transplantation may be a generalised immune response prophylactic against viral reactivation in the lymphopenic environment that may occur following AHSCT regardless of the disease.

In general, we found that anti-inflammatory cytokines were similar between MS and healthy controls at pre-AHSCT in contrast to previous reports (38, 39). This may be due to the high proportion of patients in our cohort receiving Natalizumab [as previously reported (53)], which is known to alter immunosuppressive Treg populations in MS (70). These anti-inflammatory cytokines were generally reduced following transplant except for IL-10 at day 14 in MS patients. Because anti-inflammatory cytokines are often secreted as a negative feedback response towards inflammation (23), and can contradictorily act as markers of inflammation themselves (71), their significant reduction post-AHSCT may be an indication that between 3, 6, 12, and 24 months post-AHSCT, the inflammatory responses induced by Th1, Th17 and/or acute phase inflammatory cytokines are less active than they were before transplantation in MS patients. Additionally, the significant reduction of IL-2 early after transplant suggests that previously reported Treg reconstitution (53, 59, 72–75) may not be reliant on this cytokine (76), and may instead occur as a result of lymphopenia-induced homeostatic proliferation (10). These results suggest that MS pathogenesis relates to an over-represented proinflammatory milieu, rather than a deficiency of anti-inflammatory cytokines.

Importantly, IL-17 was significantly increased at 12 months post-AHSCT in the relapse cohort compared to non-relapse patients. Overall, relapse patients did not show significant suppression of the proinflammatory Th17 cytokines IL-17, IL-23, IL-21 and IL-1β post-AHSCT, unlike the non-relapse cohort. Interestingly, the occurrence of clinical relapses ranged from 11-13 months after transplant, approximately when IL-17 was shown to be elevated, to later at 22-23 months post-AHSCT. These results suggest that failure to suppress Th17-mediated responses within the first 12 months can induce immediate to long-term clinical relapses in MS patients following AHSCT. Although not statistically significant, Th17 cytokines consistently display a transiently increasing trend at day 14 post-AHSCT in relapsed patients **(** **Figure 3** **)**. However, no conclusions could currently be drawn from this result due to a low sample size at this timepoint within the relapse cohort. Further studies would be required to confirm these the mechanisms behind these findings. For instance, suppression of pathogenic T cells in MS patients may be affected by varying rates of ATG clearance by patients (77). It may be possible to further explore the relationship between post-AHSCT clinical relapses and blood ATG clearance flow cytometrically (78), in future AHSCT MS studies.

The limitations of this study are the relatively small numbers of patient cohorts and controls, and the lack of ATG and use of more intensive immune chemotherapy in the control group. Additionally, this study did not include data for B/T cell immune reconstitution following AHSCT. Nevertheless, this study represents the first significant analysis of cytokine expression in MS patients undergoing AHSCT using BEAM/ATG.

In summary, we demonstrate that AHSCT significantly suppresses Th1- and Th17-related inflammatory cytokines in MS patients for up to 24 months. Additionally, our results support the importance of suppressing these Th17-proinflammatory responses to achieve long-term remission in MS patients but this requires validation with a larger cohort and prospective study. The suppression of Th17 proinflammatory cytokines in MS patients may be conducive to the regeneration of several immunoregulatory cell populations, including Tregs, as these cytokines are known to inhibit this process (79, 80). Future studies are required to investigate the relationship between these post-AHSCT cytokine profiles to the reconstitution of immune cell populations to further elucidate the mechanisms of immunological tolerance regeneration in MS patients following transplantation.

## Data Availability Statement

The raw data supporting the conclusions of this article will be made available by the authors, without undue reservation.

## Ethics Statement

The studies involving human participants were reviewed and approved by St Vincent’s Hospital HREC (File number 10/135). The patients/participants provided their written informed consent to participate in this study.

## Author Contributions

KH, MK, and JCM designed the research methodology. KH and MV performed the experiments. KH, MV, MK, JCM, DM, BW, and JJM analyzed the data. KH wrote the manuscript. DM, IS, and JJM designed the study. JCM and IS reviewed clinical status of MS patients. JJM, DM, and KH provided funding for the study. All authors reviewed and edited the final manuscript. All authors contributed to the article and approved the submitted version.

## Funding

This study was funded by an NHMRC Postgraduate Scholarship (RG161591), an AMR Translational Research Grant from the St Vincent’s Clinic Foundation, Reset Australia, an Arrow Bone Marrow Transplant Foundation Special Research Grant, the SVH Haematology Research Fund, Maple-Brown Family Foundation, the John Kirkpatrick Family Foundation, NSW Health, and Multiple Sclerosis Research Australia.

## Conflict of Interest

DM has received a research grant from Phebra Pty Ltd outside of submitted work. JCM reports personal fees from Biogen, Merck, Sanofi Genzyme and Teva, outside the submitted work.

The remaining authors declare that the research was conducted in the absence of any commercial or financial relationships that could be construed as a potential conflict of interest.

## Publisher’s Note

All claims expressed in this article are solely those of the authors and do not necessarily represent those of their affiliated organizations, or those of the publisher, the editors and the reviewers. Any product that may be evaluated in this article, or claim that may be made by its manufacturer, is not guaranteed or endorsed by the publisher.
